# ZNF452 facilitates tumor proliferation and invasion via activating AKT-GSK3β signaling pathway and predicts poor prognosis of non-small cell lung cancer patients

**DOI:** 10.18632/oncotarget.16408

**Published:** 2017-03-21

**Authors:** Xiupeng Zhang, Haijing Zhou, Yong Zhang, Lin Cai, Guiyang Jiang, Ailin Li, Yuan Miao, Qingchang Li, Xueshan Qiu, Enhua Wang

**Affiliations:** ^1^ Department of Pathology, College of Basic Medicine Science and First Affiliated Hospital of China Medical University, Shenyang, China; ^2^ Department of Pathology, Cancer Hospital of China Medical University, Shenyang, China; ^3^ Department of Radiotherapy, First Affiliated Hospital of China Medical University, Shenyang, China

**Keywords:** ZNF452, NSCLC, AKT signaling, proliferation, invasion and metastasis

## Abstract

ZNF452 is a zinc-finger protein family member which contains an isolated SCAN (SRE-ZBP, CTfin51, AW-1 and Number 18 cDNA) zinc-finger domain. Despite the SCAN N-terminus domain is known to play a role in transcriptional regulation of genes involved in cell survival and differentiation, there are no precise cellular functions that have been assigned to ZNF452. In the present study, we found that either endogenous or exogenous ZNF452 was overexpressed in the cytoplasm of NSCLC cells and positive ratio of ZNF452 in NSCLC samples (50.8%, 93/183) was significantly higher than that in normal lung tissues (22.4%, 13/58, P<0.001). ZNF452 overexpression was correlated with advanced TNM stage (P=0.033), positive lymph node metastasis (P=0.002) and predicted poor overall survival of NSCLC patients (P<0.001). ZNF452 facilitated tumor growth, colony formation, G1-S phase arrest, migration and invasion through upregulating the levels of CyclinD1, CyclinE1, p-Rb, or Snail, and downregulating the expression of Zo-1. In nude mice xenografts, overexpressing ZNF452 also promoted tumor proliferation and metastasis. Subsequently, we found that the effect of ZNF452 on facilitating tumor proliferation and invasion was through activating its downstream AKT-GSK3β signaling pathway. Treatment of AKT inhibitor markedly prevented the phosphorylation of AKT and GSK3β which subsequently counteracted increasing expression of CyclinD1, CyclinE1 or Snail and restored the decreasing expression of Zo-1, as well as the upregulation of tumor proliferation and invasion, caused by ZNF452 overexpression.

Taken together, the present study indicated that ZNF452 may be an upstream regulator of AKT-GSK3β signaling pathway and facilitates proliferation and invasion of NSCLC.

## INTRODUCTION

ZNF452 (also known as SCAND3) contains a SCAN (SRE-ZBP, CTfin51, AW-1 and Number 18 cDNA) zinc-finger (ZNF) domain at the N-terminus, an INT core at the flank of the N-terminus and a TPase-derived hATd dimerization module [1]. SCAN zinc-finger domain often co-exists with the KRAB (Krüppel-associated box) domain in diverse transcription factors, and they are often seen as functional domains in the C2H2-type ZNF family [2, 3]. Recent studies showed that C2H2 ZNF proteins played important roles in cancer progression through regulating transcription of downstream genes, which were involved in proliferation, apoptosis, migration and invasion [4]. Interestingly, existing literatures showed that ZNFs played different roles in different cancer types, some functioned as oncogenes and some functioned as tumor suppressors [5–12]. However, except SCAN domains, ZNF452 does not contain C2H2 ZNF domains or KRAB domains. Despite the SCAN N-terminus domain is known to play a role in transcriptional regulation of genes involved in cell survival and differentiation, there are no precise cellular functions have been assigned to ZNF452 [2–3, 13].

In this study, we explored the protein level and subcellular distribution of ZNF452 in both lung cancer tissues and cell lines, as well as their clinicopathological relevances. We also investigated the effects of ZNF452 on tumor proliferation and invasiveness after transfected with ZNF452 plasmid or ZNF452-siRNA. In conclusion, we identified that ZNF452 enhanced proliferation and invasion of NSCLC cells through facilitating the activation of AKT-GSK3β signaling pathway.

## RESULTS

### The expression of ZNF452 in NSCLC specimens

**Figure 1:**
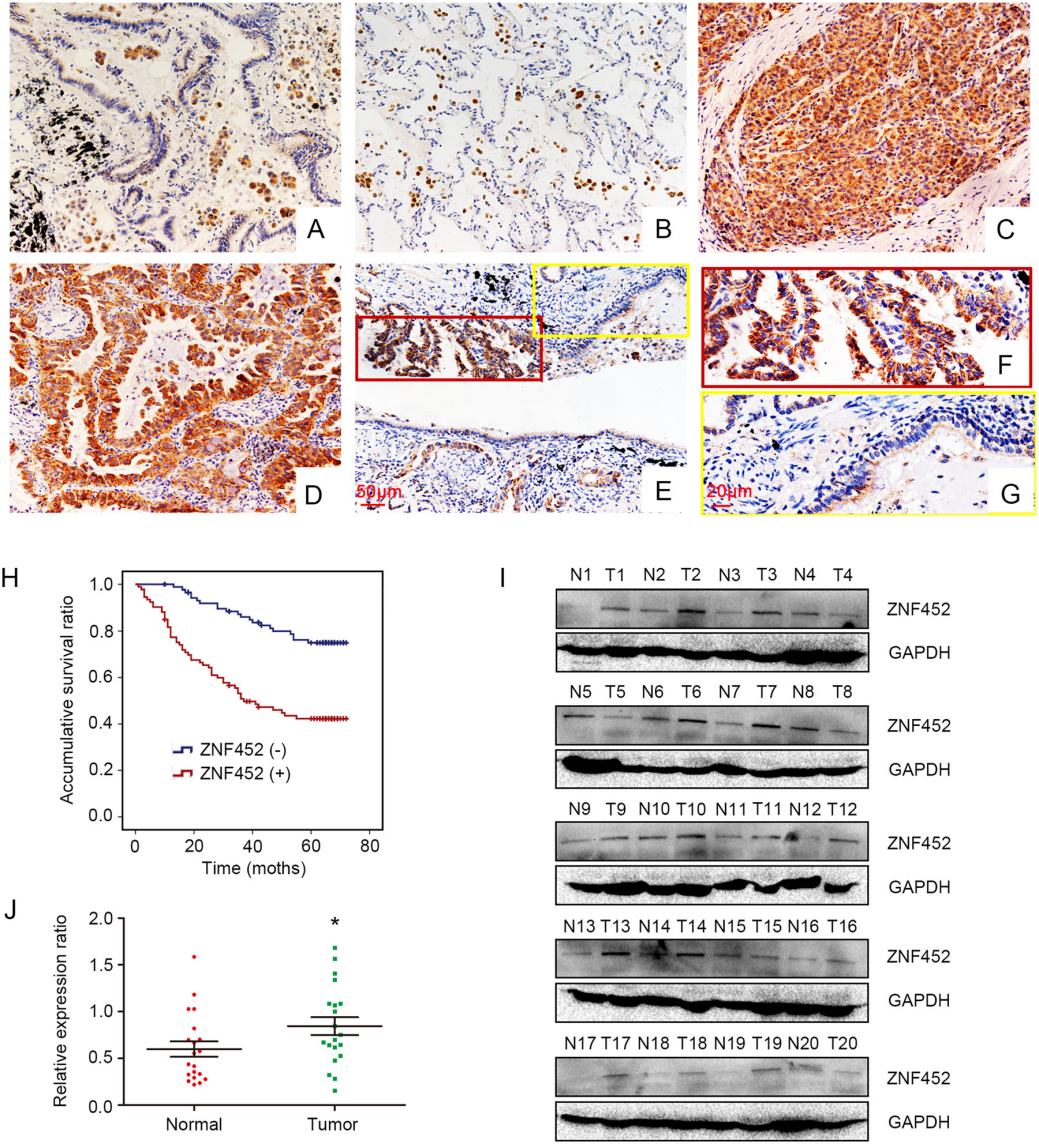
ZNF452 expression in NSCLC specimens ZNF452 was negatively or weakly expressed in the paired normal bronchial (200×, **(A)**) and alveolar epithelial cells (200×, **(B)**). ZNF452 was strongly expressed in the cytoplasm of lung cancer tissues (200×; **(C)**, squamous carcinoma; **(D)**, adenocarcinoma). The expression of ZNF452 was higher in NSCLC samples than in the corresponding noncancerous tissues (200×, **(E)**, scale bar = 50 μm; 400×, inset, **(F)** and **(G)**, scale bar = 20 μm). Kaplan-Meier analysis showed that the overall survival of NSCLC patients with ZNF452 overexpression was significantly shorter than those without ZNF452 expression **(H)**. The normalized protein levels of ZNF452 in fresh NSCLC specimens were dramatically higher than that in paired noncancerous tissues **(I-J)**.

Initially, we evaluated ZNF452 expression in 183 cases NSCLC samples and 58 cases corresponding noncancerous tissues using IHC staining. We found that ZNF452 was dimly expressed in normal lung tissues (Figure 1A-1B) but showed strong expression in the cytoplasm of NSCLC specimens (Figure 1C-1D). The positive ratio of ZNF452 expression in NSCLC (50.8%, 93/183) was significantly higher than that in normal lung tissues (22.4%, 13/58, *P*<0.001, Figure 1E-1G). Statistical analysis indicated that ZNF452 overexpression was obviously correlated with advanced TNM stage (*P*=0.033) and positive lymph node metastasis (*P*=0.002). There was no significant association between ZNF452 overexpression and sex, age, histological type or histological differentiation (Table 1). As the prognostic biological meaning or definition of tumor differentiation is different between lung adenocarcinoma and squamous cell carcinoma, we also investigated the associations between ZNF452 expression and histological differentiation in lung adenocarcinoma or squamous cell carcinoma separately. No significant correlation was seen either in lung adenocarcinoma or squamous cell carcinoma (Supplementary Table 1). Kaplan–Meier analysis result showed that the overall survival of patients with positive ZNF452 expression (42.73 ±2.87 months) was significantly shorter than those with negative ZNF452 expression (62.67 ±1.91 months, *P*<0.001, Figure 1H). Subsequent univariate analysis (UA) and multivariate analysis (MA) revealed that, along with positive lymph node metastasis (*P*<0.001 for UA and *P*=0.023 for MA), overexpression of ZNF452 (*P*<0.001 for UA and *P*<0.001 for MA, Table 2) could be considered as independent prognostic factors in NSCLC patients.

**Table 1:** Correlation of the cytosolic overexpression of ZNF452 with clinicopathological features in 183 cases of NSCLC

Clinicopathological factors	N	Positive	Negative	χ2	*P*
Age (years)					
<60	72	40	32	1.065	0.364
≥60	111	53	58		
Gender					
Male	112	61	51	1.534	0.228
Female	71	32	39		
Histological type					
Squamous cell carcinoma	66	30	36	1.201	0.549
Adenocarcinoma	115	62	53		
Large cell carcinoma	2	1	1		
Differentiation					
Well	75	36	39	0.404	0.550
Moderate+Poor	108	57	51		
TNM classification					
I	67	27	40	4.681	0.033
II+III	116	66	50		
Lymph node metastasis					
Positive	85	54	31	10.259	0.002
Negative	98	39	59		

**Table 2:** Univariate and multivariate regression analysis of the association between clinicopathological features and overall survival in 183 cases of non-small cell lung cancer (NSCLC)

Clinicopathological feature	Hazard ratio	*P*
	(95% CI)	
**Univariate analysis**		
Age	0.903(0.565-1.443)	0.903
Gender	1.010(0.632-1.614)	0.966
Histological type	1.552(0.967-2.492)	0.069
Differentiation	1.651(1.012-2.691)	0.044
TNM classification	4.580(2.407-8.715)	<0.001
Lymph node metastasis	5.001(2.949-8.481)	<0.001
ZNF452 expression	3.351(2.015-5.572)	<0.001
**Multivariate analysis**		
Differentiation	1.189(0.718-1.969)	0.501
TNM classification	2.020(0.809-5.043)	0.132
Lymph node metastasis	2.462(1.133-5.347)	0.023
ZNF452 expression	2.594(1.534-4.386)	<0.001

Then, we performed WB to test ZNF452 protein levels in 20 cases paired fresh NSCLC samples. The normalized protein level of ZNF452 in lung cancer (0.84±0.096) was obviously higher than that in noncancerous tissues (0.60±0.083, P=0.031, Figure 1I-1J).

### ZNF452 expression and localization in NSCLC cell lines

**Figure 2:**
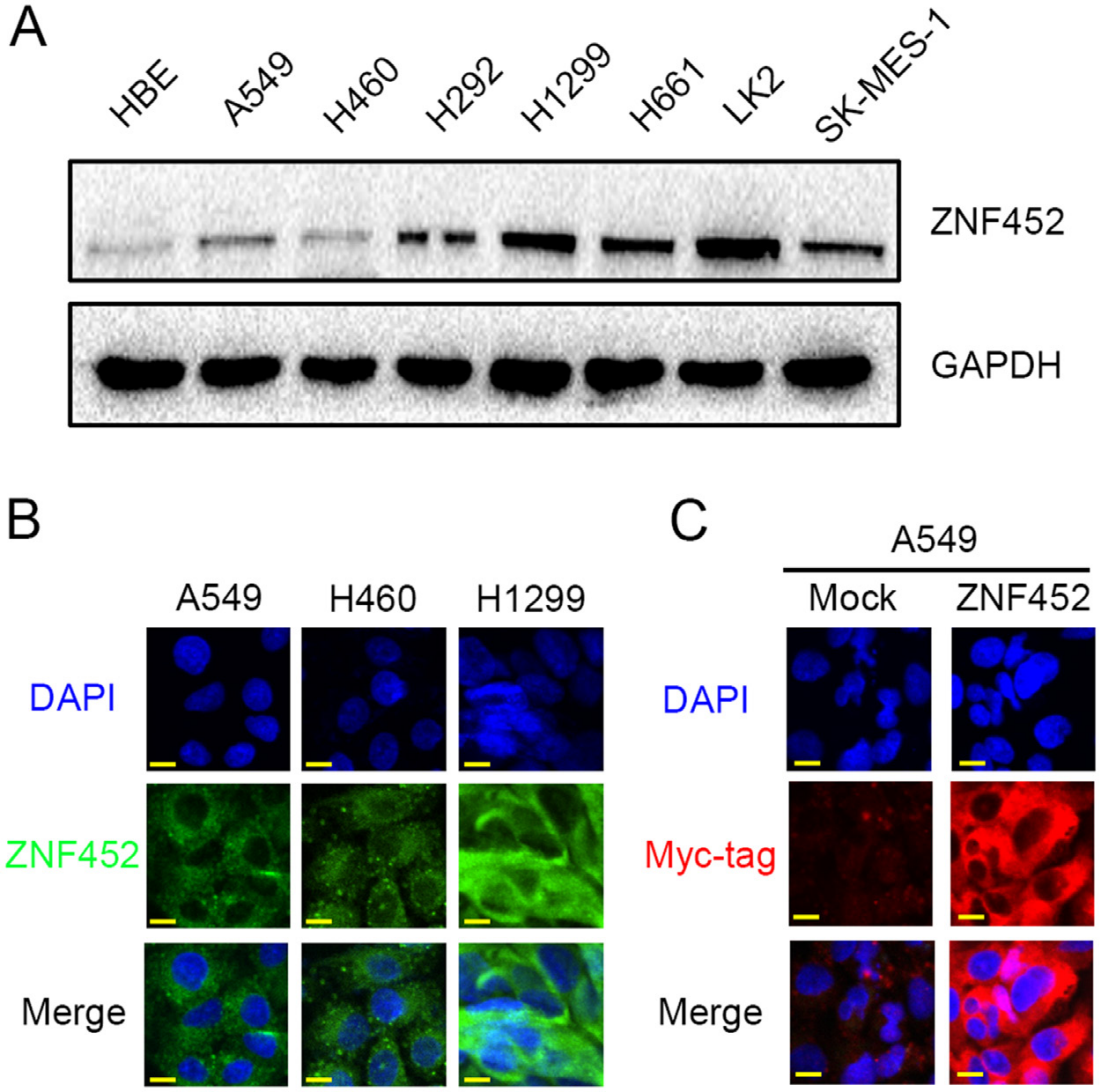
The expression and subcellular localization of ZNF452 in NSCLC cells Western blotting results showed that ZNF452 expression was higher in lung cancer cells than in HBE cells **(A)**. In A549, H460 and H1299 cells, endogenous ZNF452 localized in the cytoplasm, 600× magnification **(B)**. Using antibody of Myc-tag, we found that exogenous ZNF452 also localized in the cytoplasm of A549 cells, whereas no positive signal was detected in non-transfected cells, 600× magnification **(C)**.

We evaluated ZNF452 expression and subcellular localization in NSCLC cell lines. ZNF452 was expressed in all the 8 cell lines and 6 of 7 NSCLC cell lines which showed higher ZNF452 expression than normal bronchial epithelial cells (HBE, Figure 2A). In A549, H460 and H1299 cells, endogenous ZNF452 localized in the cytoplasm (Figure 2B). After overexpressing ZNF452 in A549 cells, we found that exogenous ZNF452 also localized in the cytoplasm (Figure 2C).

### ZNF452 promoted NSCLC cells proliferation and accelerated cell cycle progression

**Figure 3:**
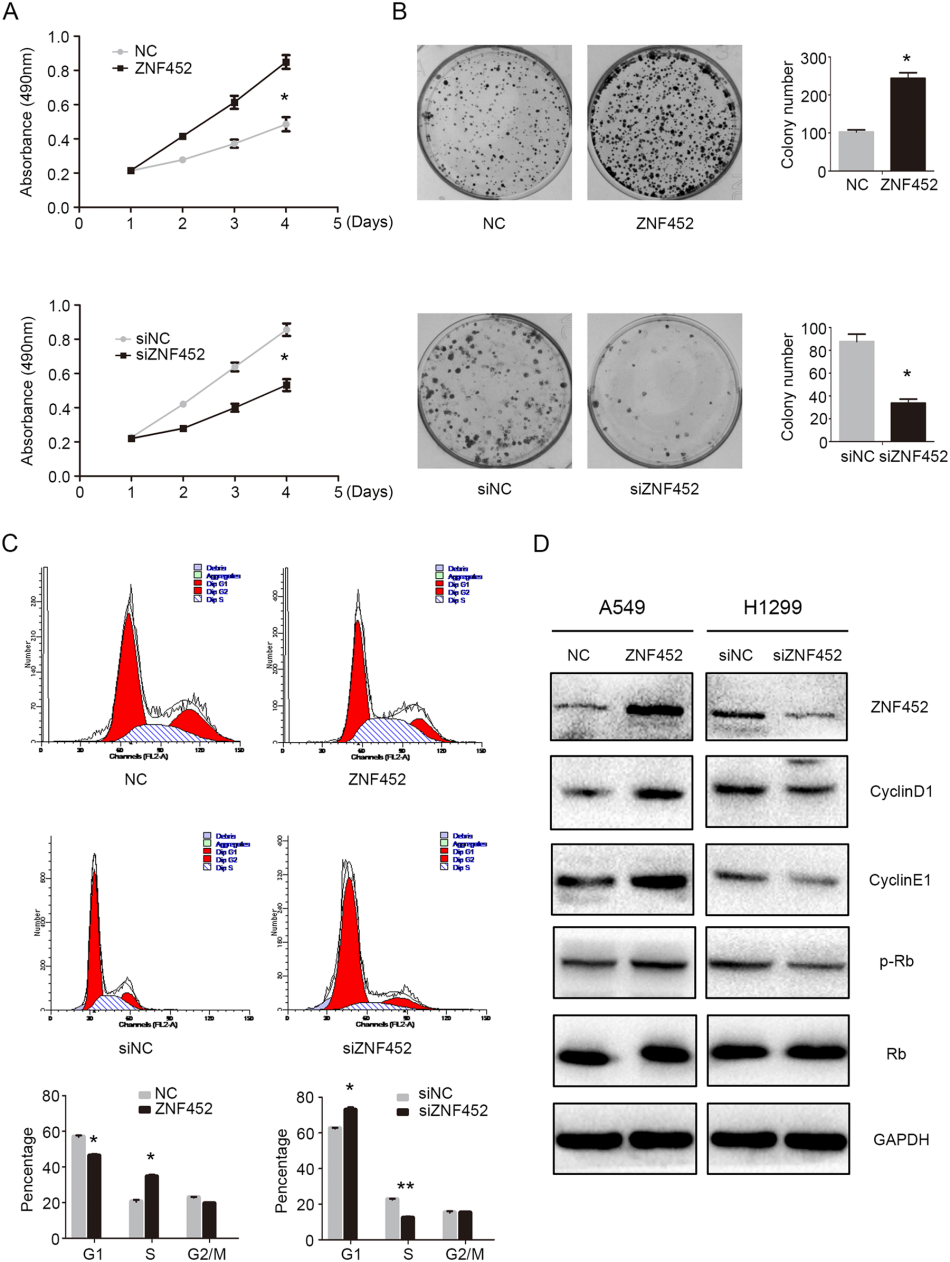
ZNF452 accelerated NSCLC cell proliferation The proliferation **(A)** and colony formation ability **(B)** were enhanced after overexpressing ZNF452 in A549 cells or depressed after depleting ZNF452 in H1299 cells. The results of flow cytometry identified that G1 phase was increased and S phase was decreased after overexpressing ZNF452, whereas G1 phase was depressed, S enhanced followed by ZNF452 knock-down. G2 phase and M phase showed no visible changes after either ZNF452 overexpression or depletion **(C)**. Subsequet western blotting results showed that CyclinD1, CyclinE1 and the phosphorylation of RB (Ser807) were increased after overexpressing ZNF452 in A549 cells or decreased after depleting ZNF452 in H1299 cells **(D)**. *P<0.05, **P<0.01.

After overexpressing ZNF452 in A549 and depleting ZNF452 in H1299 cells, MTT and colony formation assay results suggested that upregulating ZNF452 expression enhanced tumor growth and colony formation abilities in A549 cells, whereas downregulating ZNF452 expression depressed tumor growth and colony formation abilities in H1299 cells (Figure 3A-3B). Flow cytometry analysis was employed to characterize the cell cycle status in A549 cells with ZNF452 plasmid transfecting or in H1299 cells with ZNF452-siRNA transfection. We found that G1 phase was decreased and S phase were increased after transfecting ZNF452 plasmids in A549 cells; however, G1 phase was enhanced and S phase was depressed followed by depletion of ZNF452 with siRNA in H1299 cells (Figure 3C). The G2/M phase showed no visible changes after either upregulating or downregulating the protein levels of ZNF452 (Figure 3C). Subsequently, the protein levels of cell cycle related molecules were also examined by western blot followed by overexpressing ZNF452 in A549 cells or inhibiting ZNF452 in H1299 cells. The protein levels of CyclinD1 and CyclinE1, as well as p-Rb, were upregulated after ZNF452 overexpression, and they were correspondingly decreased by ZNF452 knock-down (Figure 3D). The other cyclin proteins showed no obvious changes either after upregulating or downregulating the protein levels of ZNF452 (Supplementary Figure 1).

### ZNF452 increased NSCLC cells migration and invasion

**Figure 4:**
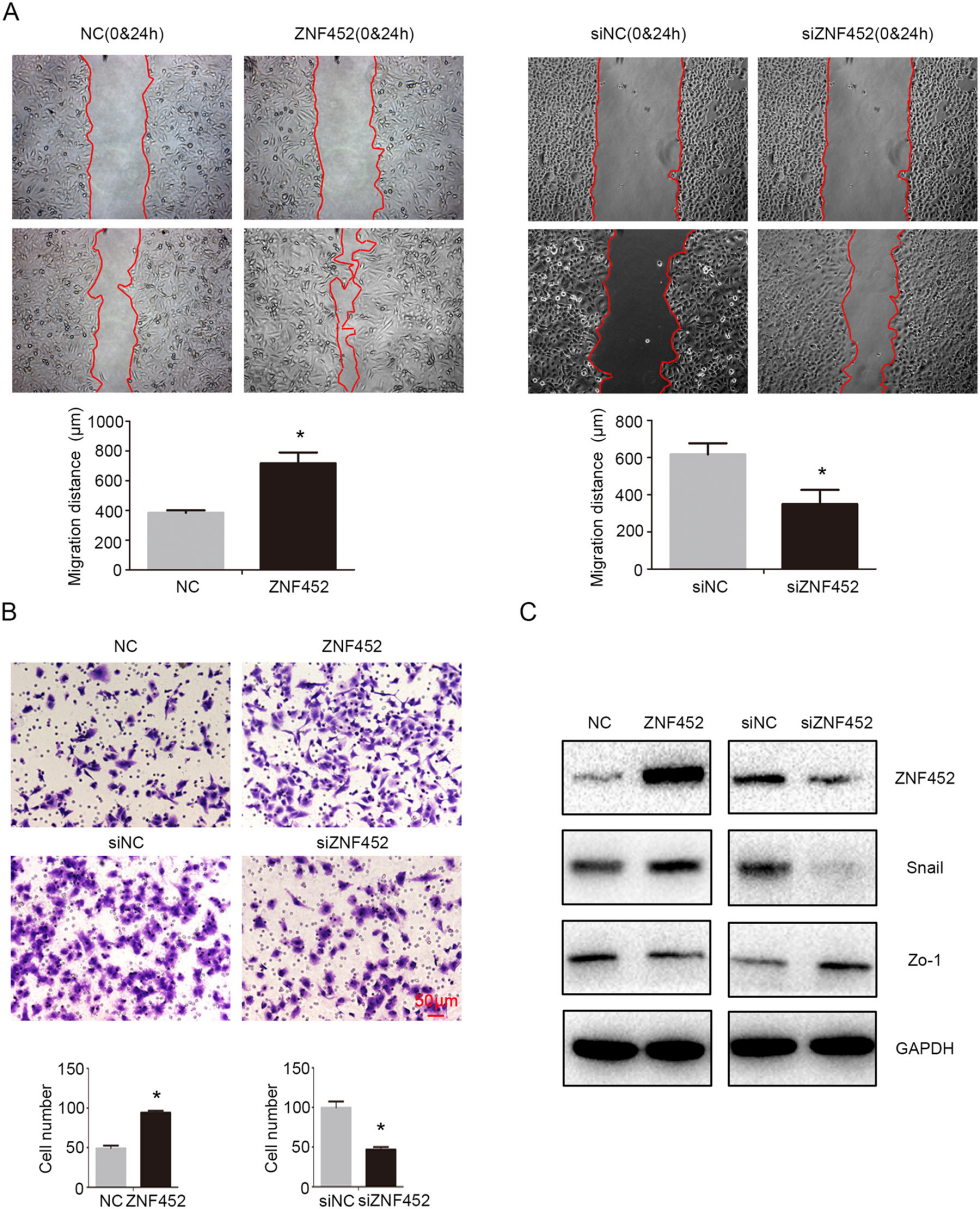
ZNF452 promoted migration and invasion of NSCLC cells Tumor migration **(A)** and invasion (**(B)**, scale bar = 50 μm) were also enhanced after overexpressing ZNF452 in A549 cells or depressed after depleting ZNF452 in H1299 cells. Snail was upregulated and Zo-1 was downregulated after transfecting ZNF452 plasmid, whereas Snail was downregulated and Zo-1 was upregulated followed by depleting ZNF452 in H1299 cells **(C)**. *P<0.05.

Tumor migration (Figure 4A) and invasion (Figure 4B) were also enhanced by transfecting ZNF452 plasmid in A549 cells or depressed by transfecting ZNF452 siRNA in H1299 cells. Western blotting results revealed that Snail was increased and Zo-1 was decreased after overexpressing ZNF452 in A549 cells (Figure 4C). Accordingly, Snail was upregulated and Zo-1 was downregulated followed by depleting ZNF452 in H1299 cells (Figure 4C). Other proteins such as Slug, Occludin, E-cadherin, α-catenin, N-cadherin and Vimentin presented no visible alterations (Supplementary Figure 2).

### ZNF452 accelerated NSCLC malignant phenotype *in vivo*

**Figure 5:**
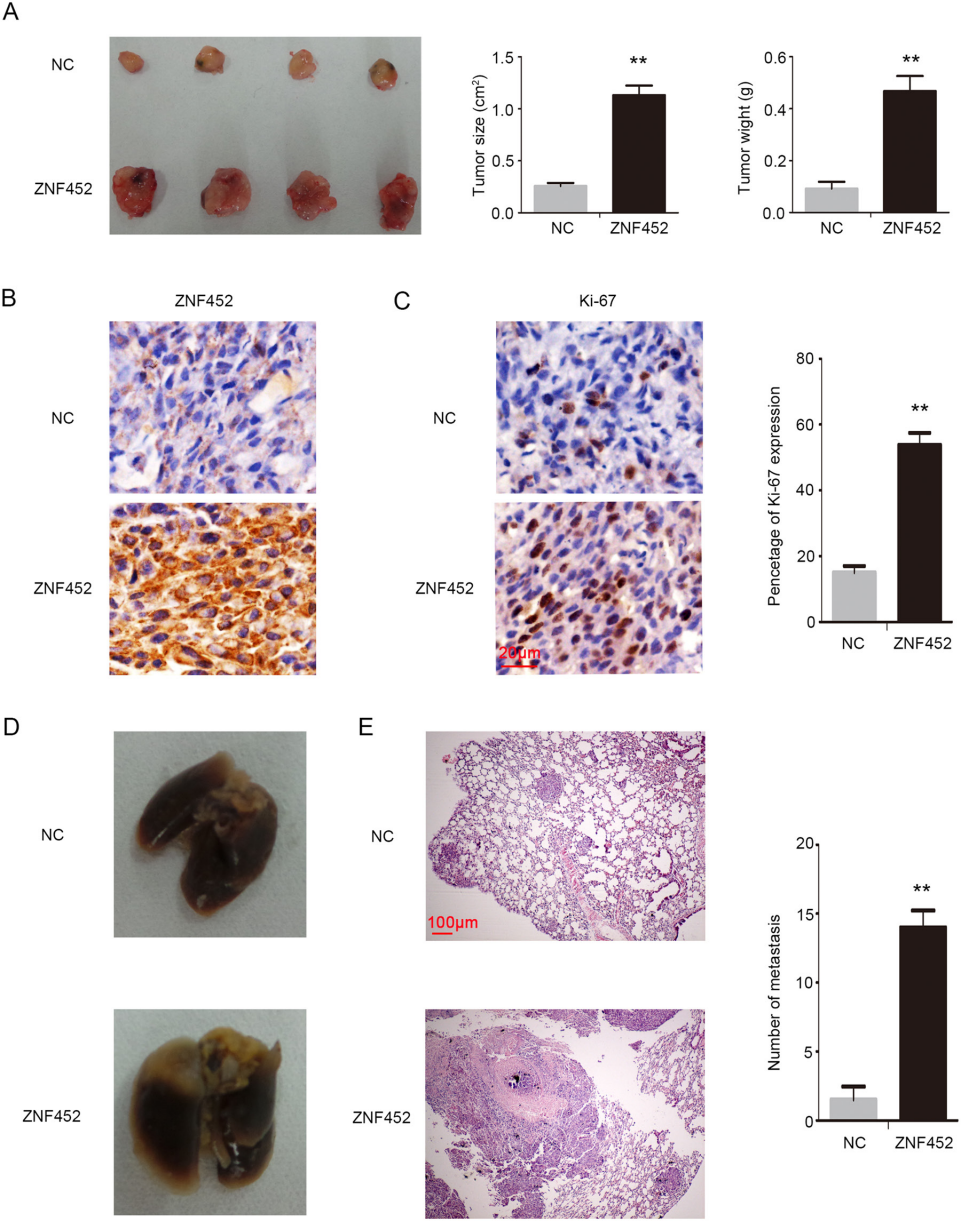
ZNF452 enhanced proliferation and invasion of NSCLC cells *in vivo* In the xenograft assay (left), the tumor volume (middle) and weight (right) in ZNF452 overexpressing group were significantly higher than in the control group **(A)**. ZNF452 presented visible strong cytosolic expression in the ZNF452-A549^(+)^ group, however, the control group showed negative or weak cytosolic expression, 400×, (**B**, scale bar = 20 μm). Ki-67 index was also dramatically higher in the ZNF452-A549^(+)^ group than in the control group, 400×, (**C**, scale bar = 20 μm). Mice injected with A549-ZNF452 ^(+)^ through the tail vein developed more pulmonary metastases **(D)** than the control group, 40×, (**E**, scale bar = 100 μm). **P<0.01.

In order to evaluate the effect of ZNF452 on tumor proliferation *in vivo*, we subcutaneously injected ZNF452-transfected A549 cells (A549-ZNF452 ^(+)^) or control cells into the axillae of nude mice. The average tumor volume and weight or ZNF452 expression in A549-ZNF452^(+)^ group were significantly higher than in the control group (Figure 5A-5B). The Ki-67 expression was higher in ZNF452 overexpressing group than the control (Figure 5C). In order to evaluate the effect of ZNF452 on tumor metastasis *in vivo*, we injected ZNF452-transfected A549 cells or control cells into the tail vein of nude mice. In the group that received tail vein injections, the A549-ZNF452 ^(+)^group had a higher lung metastatic ratio (4/4) than the control group (2/4) and a significantly larger number of lung metastatic nodules (*P*<0.01; Figure 5D–5E).

### ZNF452 promoted phosphorylation of AKT and GSK3β

**Figure 6:**
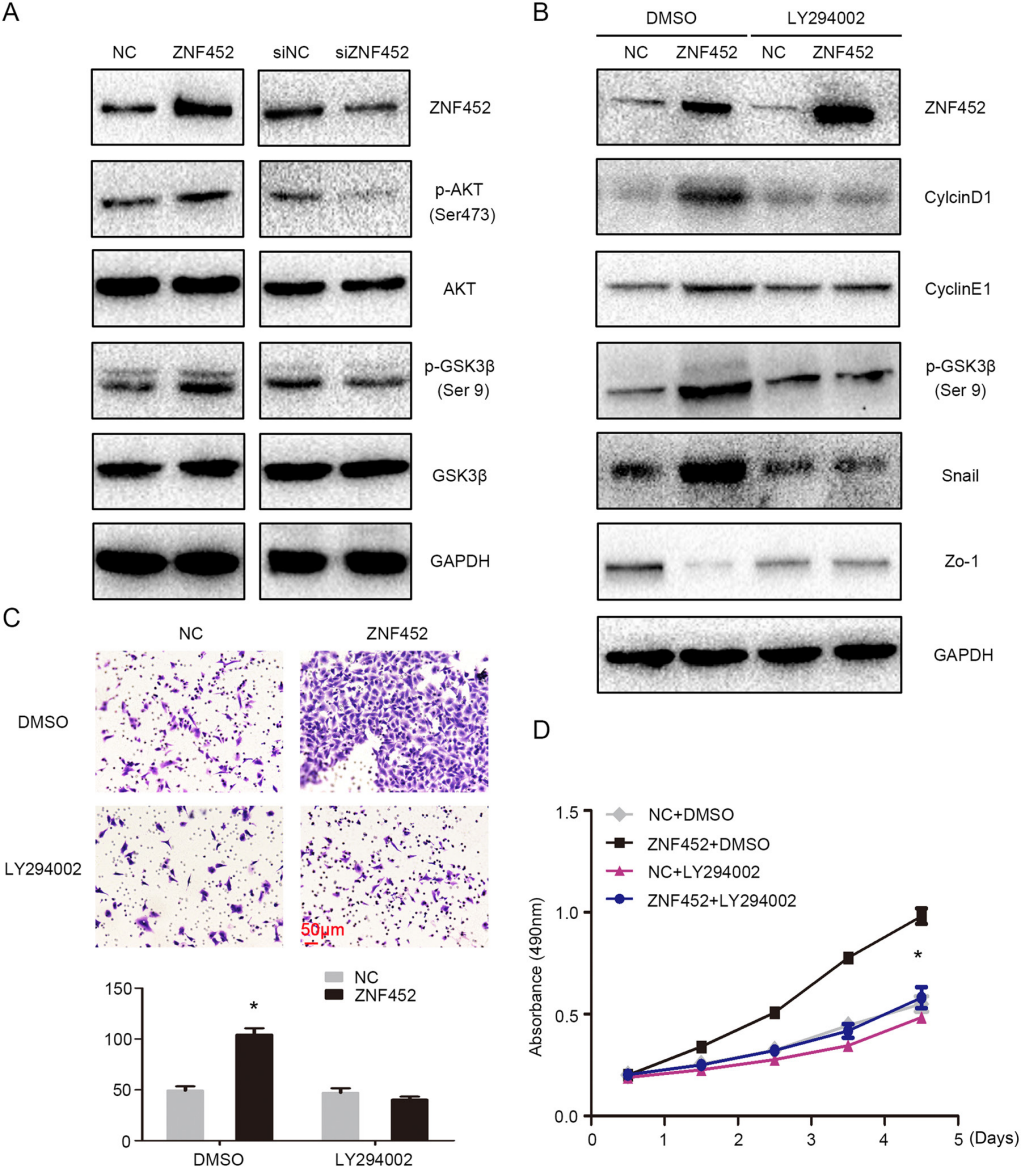
ZNF452 activated AKT signaling pathway The phosphorylation of AKT and GSK3β were increased after overexpressing ZNF452 in A549 cells and were depressed after depleting ZNF452 in H1299 cells **(A)**. The upregulation of CyclinD1, CyclinE1 or Snail and the downregulation of Zo-1 caused by ZNF452 overexpression were counteracted by incorporating LY294002, an AKT inhibitor. **(B)** The increasing of tumor proliferation **(C)** and invasion (**(D)**, scale bar = 50 μm) by ZNF452 overexpression were also reversed after adding AKT inhibitor. *P<0.05.

Finally, we screened the key signaling pathway factors involved in accelerating lung cancer proliferation and invasion. The levels of p-AKT (Ser 473) and its downstream protein, p-GSK3β (Ser 9), were upregulated after overexpression of ZNF452 in A549 cells and were downregulated after depletion of ZNF452 in H1299 cells (Figure 6A). Other key signaling transduction proteins such as p-ERK, ERK, p-p38, P38, p-JNK, JNK, p-FAK, FAK, Active-β-catenin, β-catenin, p-NF-κB, and NF-κB presented no obvious changes (Supplementary Figure 3). Treatment of AKT inhibitor markedly prevented the phosphorylation of AKT and GSK3β and subsequently counteracted the increasing expression of CyclinD1, CyclinE1 or Snail and restored the decreasing expression of Zo-1 caused by ZNF452 overexpression (Figure 6B). The upregulation of tumor proliferation and invasion mediated by ZNF452 overexpression was also reversed by AKT inhibitor incorporation (Figure 6C-6D).

## DISCUSSION

Our data revealed that ZNF452 played an oncogenic role in NSCLC cells, which promoted the expression of CyclinD1, CyclinE1 and Snail, and inhibited the expression of Zo-1 through activating AKT- GSK3β signaling pathway. Furthermore, overexpression of ZNF452 associated with advanced TNM stage, positive regional lymph node metastasis and was an independent predictor of poor prognosis in NSCLC patients.

Previous studies predicted that ZNF452 expressed in almost all eutherian genomes [13]. In our study, positive ZNF452 expression was found in both lung cancer tissues/cells and noncancerous lung tissues/cells. The protein levels of ZNF452 were upregulated in NSCLC tissues/cells than that in noncancerous lung tissues/cells. As different ZNFs played different roles during carncinogenesis [5–12], we further investigated the clinicopathological associations of ZNF452 and confirmed that ZNF452 functioned as an oncogene in NSCLCs and correlated with advanced TNM staging and positive regional lymph node metastasis. ZNF proteins tended to locate in nucleus and their nuclear localization was controlled by the interaction between KRAB domain and KAP1 [14, 15]. Since there was no KRAB domain in ZNF452, our data presented similar results that ZNF452 was expressed in the cytoplasm in both NSCLC tissues /cells tested when there was no KRAB-KAP1 interaction.

ZNF family members play a central role in regulating gene expression and therefore coordinate multiple biological processes, including differentiation, development, metabolism, apoptosis, autophagy and stemness maintenance [16–21]. As a family member of ZNF lacking of C2H2 domain and KRAB domain, no precise cellular function has been directly assigned to ZNF452 but its SCAN N-terminus domain is known to play a role in transcriptional regulation of genes involved in metabolism, cell survival and differentiation [2, 3]. In the present study, we found that ZNF452 facilitated proliferation and G1-S phase arrest of lung cancer cells, *in vitro* or *in vivo*, through upregulating the levels of CyclinD1, CyclinE1 and p-Rb. Meanwhile, ZNF452 also promoted migration and invasion of lung cancer cells through upregulating the expression of Snail and inhibiting the expression of Zo-1. Our results were consistent with the previous studies that N-terminus SCAN domain alone plays a role in regulating the process of differentiation and development [2, 3]. However, Williams et al. showed that the isolated SCAN domain was not associated with either transcriptional activation or repression [22, 23]. Therefore, the underlying mechanisms of how ZNF452 facilitates the expression of key proteins involved in regulating proliferation and migration remain to be determined.

A number of ZNF family members promoted cell growth and metastasis through activating or inactivating genes involved in signaling pathway, including TGF-β, AKT and MAPK et al [24–27]. Therefore, we evaluated key proteins of common signaling pathways involved in regulating proliferation and migration in NSCLC. We found that ZNF452 facilitated the phosphorylation of AKT-GSK3β signaling pathway. Treatment of AKT inhibitor markedly prevented the phosphorylation of AKT and GSK3β and subsequently counteracted the increasing expression of CyclinD1, CyclinE1 or Snail and restored the decreasing expression of Zo-1 caused by ZNF452 cDNA transfection. The upregulation of tumor proliferation and invasion mediated by ZNF452 overexpression was also reversed by AKT inhibitor incorporation. Previous studies had shown that there may be a sumoylation site in the N-terminus of SCAN family members despite there was no direct evidence of direct interaction between them [28]. Sumoylation played important roles in regulating multiple signaling pathways such as AKT and MAPK [29, 30]. Our results raised the possibility that ZNF452 may be involved in the process of sumoylation of downstream signaling pathway, which certainly needed to be further elucidated.

In conclusion, the present study indicated that overexpression of ZNF452 correlated with advanced TNM stage, positive regional lymph node metastasis and predicted poor prognosis of NSCLC patients. ZNF452 may be the upstream regulator of AKT-GSK3β signaling pathway and facilitates proliferation and invasion of NSCLC through enhancing the phosphorylation of and AKT and GSK3β.

## MATERIALS AND METHODS

### Patients and clinical specimens

This study was approved by the local institutional review board of the China Medical University. Tissue samples were obtained from 183 patients (112 males and 71 females) who underwent complete surgical excision at the First Affiliated Hospital of China Medical University with a diagnosis of lung squamous cell carcinoma, lung adenocarcinoma or lung large cell carcinoma from 2010 to 2012. The patients who underwent neoajuvant radiotherapy and/or chemotherapy were excluded from the cohort of the present study. All patients received standard chemotherapy after surgery. Of the 183 cases, 58 cases had corresponding noncancerous tissues. All the 183 lung cancer cases contained complete follow-up data. The survival of each patient was defined as the time from the day of surgery to the end of follow-up or the day of death due to recurrence or metastasis. Histological diagnosis and grading were evaluated according to the 2015 World Health Organization (WHO) classification of tumors of lung [31]. All 183 specimens were for histological subtype, differentiation, and tumor stage. Tumor staging was performed according to the seventh edition of the Union for International Cancer Control (UICC) TNM staging system for lung cancer [32]. The median age in 183 patients was 61 years old (range from 29 years old to 80 years old). Of the 183 patients, 73 patients were older than 61 years. The samples included 66 squamous cell lung carcinoma and 115 lung adenocarcinoma and 2 large cell lung carcinoma cases, respectively. A total of 75 tumors were well differentiated, while 108 were classified as moderately or poorly differentiated. Lymph node metastases were present in 97 of the 183 cases. The tumors included 67 stages I cases and 116 stage II-III cases.

### Immunohistochemistry (IHC)

Samples were fixed in 10% neutral formalin, embedded in paraffin, and sliced in 4-μm thick sections. Immunostaining was performed by the streptavidin-peroxidase method. The sections were incubated with a monoclonal mouse anti-ZNF452 antibody (1:100; Santa cruz) at 4°C overnight, followed by biotinylated goat anti-mouse IgG secondary antibody. After washing, the sections were incubated with horseradish peroxidase-conjugated streptavidin–biotin (Ultrasensitive; MaiXin, Fuzhou, China) and developed using 3, 3-diaminobenzidine tetra-hydrochloride (MaiXin). Finally, samples were lightly counterstained with hematoxylin, dehydrated in alcohol, and mounted. Two investigators blinded to the clinical data semi-quantitatively scored the slides by evaluating the staining intensity and percentage of stained cells in representative areas. The staining intensity was scored as 0 (no signal), 1 (weak), 2 (moderate), or 3 (high). The percentage of cells stained was scored as 1 (1–25%), 2 (26–50%), 3 (51–75%), or 4 (76–100%). A final score of 0–12 was obtained by multiplying the intensity and percentage scores. Tumors were seen as positive ZNF452 expression with a score ≥4. Tumor samples with scores between 1 and 3 were categorized as showing weak expression, whereas those with scores of 0 were considered to have no expression; both weak expression and no expression were defined as negative ZNF452 expression.

### Cell culture

The HBE cell line was obtained from the American Type Culture Collection (ATCC; Manassas, VA, USA). The A549, H460, H292, H1299, H661, and SK-MES-1 cell lines were obtained from the Shanghai Cell Bank (Shanghai, China). The LK2 cell line was a gift from Dr. Hiroshi Kijima (Department of Pathology and Bioscience, Hirosaki University Graduate School of Medicine, Japan). All cells were cultured in RPMI 1640 (Invitrogen, Carlsbad, CA, USA) supplemented with 10% fetal bovine serum (Invitrogen), 100 IU/ml penicillin (Sigma), and 100 μg/ml streptomycin (Sigma), and passaged every other day using 0.25% trypsin (Invitrogen).

### Western blotting

Total protein was extracted using a lysis buffer (Pierce, Rockford, IL, USA) and quantified with the Bradford method [33]. Fifty μg of the total protein samples were separated by 10% SDS-PAGE, and transferred onto polyvinylidene fluoride membranes (PVDF; Millipore, Billerica, MA, USA). Membranes were incubated overnight at 4°C with the following primary antibodies: ZNF452 (1:100, sc-514003, Santa Cruz, CA, USA), GAPDH (1:5000, Sigma, St. Louis, MO, USA), Myc-tag, Cyclin A2, Cyclin B1, Cyclin D1, Cyclin D2, Cyclin D3, Cyclin E1, Cyclin E2, Cyclin H, p-P38, P38, p-ERK, ERK, p-AKT, AKT, p-JNK, JNK, p-FAK, FAK, RB, p-RB, p-NF-κB, p-GSK3β, Snail, Slug, Vimentin, and Active-β-catenin (1:1000; Cell Signaling Technology, Danvers, MA, USA). E-cadherin, N-cadherin, β-catenin, α-catenin, NF-κB, GSK3β, and Fibronectin(1:1000; BD Transduction Laboratories, Lexington, KY, USA), Zo-1, and Occludin (1:500; Proteintech, Chicago, IL, USA). Membranes were washed and subsequently incubated with peroxidase-conjugated anti-mouse or anti-rabbit IgG (Santa Cruz Biotechnology) at 37°C for 2h. Bound proteins were visualized using electrochemiluminescence (Pierce, Rockford, IL, USA) and detected with a bio-imaging system (DNR Bio-Imaging Systems, Jerusalem, Israel).

### Plasmid transfection and small interfering RNA treatment

Plasmids pCMV6-ddk-myc and pCMV6-ddk-myc-ZNF452 were purchased from Origene (Rockville, MD, USA).ZNF452-siRNA (sc-95338) and NC-siRNA (sc-37007) was purchased from Santa Cruz Biotechnology. Transfection was carried out using the Lipofectamine 3000 reagent (Invitrogen) according to the manufacturer's instructions.

### Immunofluorescence staining

Cells were fixed with 4% paraformaldehyde, blocked with 1% bovine serum albumin, and incubated overnight with ZNF452 and myc-tag monoclonal antibodies (1:100; Santa cruz) at 4°C. Then, the cells were incubated with tetramethylrhodamine isothiocyanate-conjugated secondary antibodies (Cell Signaling Technology) at 37°C for 2 h; cell nuclei were counterstained with 4′,6-diamidino-2-phenylindole (DAPI). Epifluorescence microscopy was performed using an inverted Nikon TE300 microscope (Nikon Co., Ltd., Tokyo, Japan), and confocal microscopy was performed using a Radiance 2000 laser scanning confocal microscope (Carl Zeiss, Oberkochen, Germany).

### MTT

Cells were plated in 96-well plates in medium containing 10% fetal bovine serum at about 3000 cells per well 24 hours after transfection. For quantitation of cell viability, cultures were stained after 4 days by using the MTT assay. Briefly, 20 μl of 5 mg/ml MTT (Thiazolyl blue) solution was added to each well and incubated for 4 hours at 37°C, then the media was removed from each well, and the resultant MTT formazan was solubilized in 150 μl of DMSO. The results were quantitated spectrophotometrically by using a test wavelength of 490 nm, each carried out in triplicate.

### Colony formation assay

The A549 and H1299 cells were transfected with pCMV6 or pCMV6- ZNF452 plasmids, negative control or ZNF452-siRNA for 48 hours. Thereafter, cells were planted into three 6-cm cell culture dishes (1000 per dish for A549 and H1299 cell lines) and incubated for 12 days. Plates were washed with phosphate buffer saline and stained with Giemsa. The number of colonies with more than 50 cells was counted. The colonies were manually counted by microscope, each carried out in triplicate.

### Flow cytometry for cell cycle analysis

Cells (500,000) were seeded into 6-cm tissue culture dishes. Twelve hours later, cells were transfected with ZNF452 plasmid or empty vector and ZNF452-siRNA or NC-siRNA. Forty-eight hours after transfection, cells were harvested, fixed in 1% paraformaldehyde, washed with phosphate-buffered saline (PBS), and stained with 5 mg/ml propidium iodide in PBS supplemented with RNase A (Roche, Indianapolis, IN) for 30 min at room temperature. Data were collected using BD systems. One-parameter histogram was plotted according to the distribution of nuclear DNA content in each cell detected by a flow cytometer. Cells in each individual phase of the cell cycle were determined based on their DNA ploidy profile.

### Matrigel invasion assay

Cell invasion assays were performed using 24-well Transwell chambers with 8-μm pores (Costar, Cambridge, MA, USA). The inserts were coated with 20μl Matrigel (1:3 dilution; BD Bioscience, San Jose, CA, USA). Cells were trypsinized 48 h after transfection, resuspended at the concentration of 3×105 cells in 100 μl of serum-free medium, and transferred to the upper chamber of Transwell plates, whereas10% FBS was added to the lower chamber as chemoattractant. After incubation for 18 h, cells that passed through the filter were fixed with 4% paraformaldehyde, stained with hematoxylin, and counted under a microscope in 10 randomly selected fields at 40× magnification.

### Wound healing assay

Wounds were created in confluent areas of cell monolayers with < 90% confluence 48 h after transfection, using a 200-μl pipette tip. Cell migration into the wound was observed at different time points and wound areas were measured using the Image J software; representative images were taken. Each condition was analyzed in duplicate, and three independent experiments were performed.

### Transplantation of tumor cells into nude mice

The nude mice used in this study were treated following the experimental animal ethics guidelines issued at China Medical University. Four-week-old female BALB/c nude mice were purchased from Slac (Shanghai, China). Mice were kept in a laminar-flow cabinet under specific pathogen-free conditions for two weeks before use. Each mouse was inoculated subcutaneously in the axilla with 5×10^6^ tumor cells or the tail vein with 2×10^6^ tumor cells (ZNF452-transfected A549 or corresponding vector-transfected control cells) in 0.2 ml sterile phosphate-buffered saline (PBS). Six weeks after inoculation, the mice were sacrificed and autopsied to examine tumor growth and dissemination. In addition, the tumor mass, heart, liver, lung, and kidney were dissected. A portion of tissue from the tumor and each organ was fixed in 4% formaldehyde (Sigma) and embedded in paraffin. Serial 6-μm-thick sections were cut and stained with H&E. The stained sections were examined microscopically.

### Statistical analysis

SPSS version 22.0 for windows (SPSS, Chicago, IL, USA) was used for all analyses. The Pearson's Chi-square test was used to assess possible correlations between ZNF452 and clinicopathological factors. Kaplan–Meier survival analyses were carried out in 183 cases specimens and compared using the log-rank test. The Cox regression model was used to test the prognostic value. All of the clinicopathological parameters were included in the Cox regression model and tested by univariate analysis and multivariate analysis using the enter method. Mann-Whitney U test was used for the image analysis of western blot results and the invasive assay results. P<0.05 was considered to indicate statistically significant differences.

## SUPPLEMENTARY MATERIALS FIGURES AND TABLES


