# Supplementary material for: ZNF452 facilitates tumor proliferation and invasion via activating AKT-GSK3β signaling pathway and predicts poor prognosis of non-small cell lung cancer patients
Source: Oncotarget. 2017 Mar 21;8(24):38863–75. doi: 10.18632/oncotarget.16408 (PMC5503578; doi:10.18632/oncotarget.16408)
Supplement: Supplementary file 1 [file oncotarget-08-38863-s001.pdf]

ZNF452 facilitates tumor proliferation and invasion via activating AKT-GSK3β signaling pathway and predicts poor prognosis of non-small cell lung cancer patients

SUPPLEMENTARY FIGURES AND TABLE

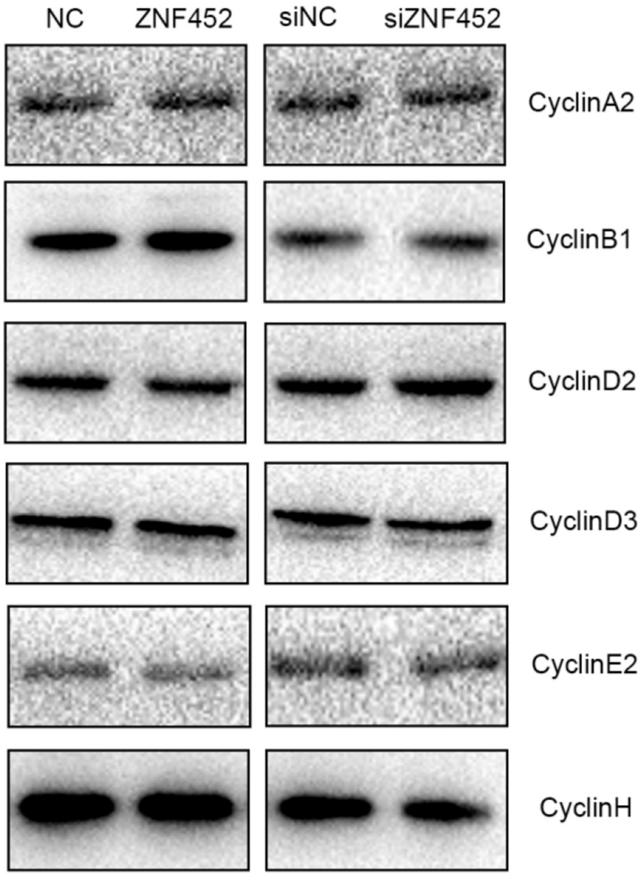

Supplementary Figure 1: ZNF452 enhance NSCLC proliferation. CyclinA2, CyclinB1, CyclinD2, CyclinD3, CyclinE2, and CyclinH revealed no significant changes after overexpressing or interfering ZNF452.

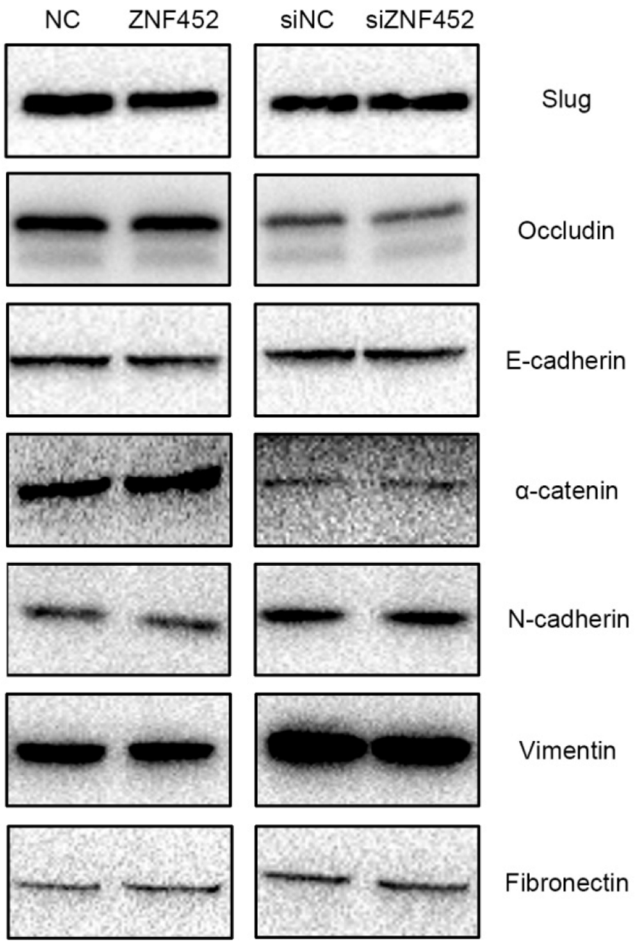

Supplementary Figure 2: ZNF452 enhance NSCLC invasion and metastasis. Slug, Occludin, E-cadherin, α-catenin, N-cadherin, Vimentin and Fibronectin demonstrated no significant changes after overexpressing or interfering ZNF452.

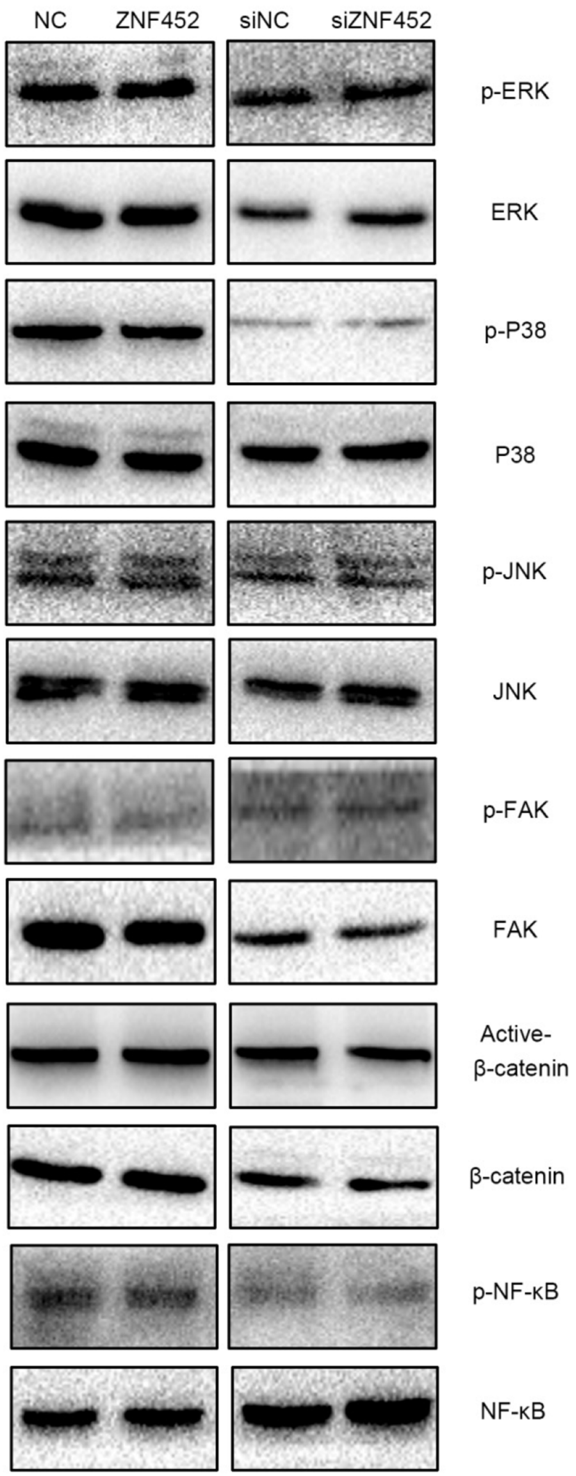

Supplementary Figure 3: ZNF452 activating phosphorylation of AKT and GSK3β. P-ERK,p-P38,p-JNK, active-β-catenin, p-FAK and p-NF-κB demonstrated no significant changes after overexpressing or interfering ZNF452.

Supplementary Table 1: The correlation between ZNF452 expression and histological differentiation in different type of NSCLC

Histological type	N	ZNF452		$\chi 2$	*P*
		Positive	Negative		
Adenocarcinoma differentiation					
Well	59	31	28	0.092	0.852
Moderate+Poor	56	31	25		
Squamous cell carcinoma differentiation					
Well	14	4	10	2.043	0.228
Moderate+Poor	52	26	26		
